# The Mystery of Electrical Storm: A Case Report

**DOI:** 10.7759/cureus.57202

**Published:** 2024-03-29

**Authors:** Imaad Rahman, Muhammad Sohail

**Affiliations:** 1 Emergency Department, East Lancashire Teaching Hospitals, Blackburn, GBR

**Keywords:** electrolytes imbalance, structural heart, out of hospital cardiac arrest, antiarrhythmic, ventricular tachycardia (vt) storm

## Abstract

Electrical storm is a cardiac emergency, defined as three or more hemodynamically unstable ventricular tachyarrythmias within 24 hours or ventricular tachycardia reoccurring within five minutes. The trigger for an electrical storm can be reversible like drug toxicity and electrolyte imbalances or can be irreversible like structural heart disease. Symptomatic patients can have chest pain, palpitations or syncopal episodes. We present a case of a gentleman in his 60s who was diagnosed with electrical storm which started as an out-of-hospital cardiac arrest. Uniqueness in the case lies in the unknown aetiology after all the investigations came back as normal and management of such cases is based on pacemakers and use of antiarrythmic agents to control and prevent further attacks.

## Introduction

Ventricular tachycardia (VT) is defined as a cardiac arrhythmia with three or more consecutive complexes arising from the ventricle at a rate of more than 100 beats per minute [1]. Ventricular storm is defined as three or more episodes of sustained ventricular tachycardia within a 24-hour period, each requiring termination with chemical or electrical cardioversion [2]. Management is twofold with acute management and prevention of relapse [3]. Most VT storms occur in patients with structural heart disease which have a low ejection fraction. However it can also occur in structurally normal hearts due to various causes like electrolyte abnormalities, toxicosis, sympathetic overdrive or congenital causes [4]. Management of electrical storm is difficult and poses a huge challenge, requiring a multidisciplinary approach for an effective treatment. Various management strategies like antiarrhythmic drugs, electrolyte abnormalities correction and placement of implantable cardioverters are common [5]. It is difficult to identify a specific cause for electrical storms and hence the mortality is high in such cases [6].

## Case presentation

**Figure 1:**
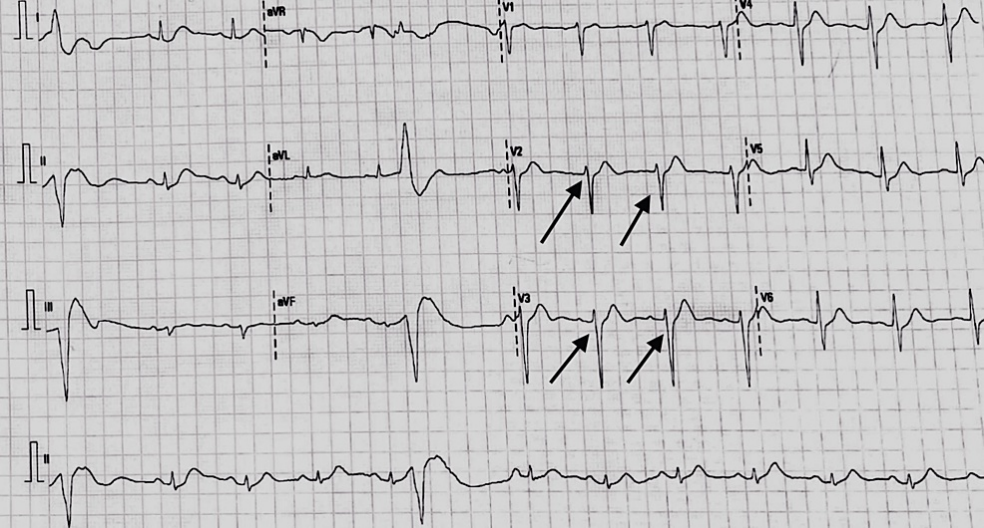
Electrocardiogram (ECG) post cardiac arrest in emergency department: shows normal sinus rhythm (arrows showing narrow QRS complexes, regular RR intervals, P waves present)

**Figure 2:**
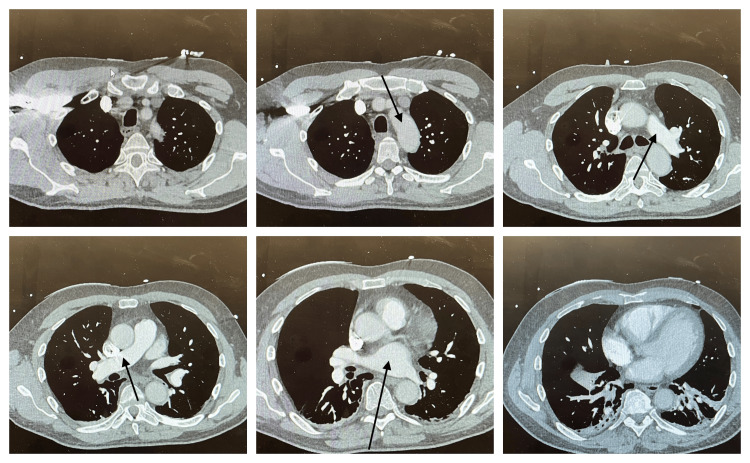
Computed tomography (CT) pulmonary angiogram done to rule out pulmonary embolism (arrows showing no evidence of embolus in the pulmonary arteries)

A gentleman in his 60s presented to a general physician with a six-week history of dry cough. He fainted at GP surgery and was found to be in cardiac arrest. He was revived using cardiopulmonary resuscitation (CPR) and an automated external defibrillator (AED). Post-revival ECG was normal, and he was asymptomatic. On being transferred to the emergency department, he was closely observed and had another cardiac arrest which was converted back to sinus rhythm using shock. He was asymptomatic with no chest pain, shortness of breath or palpitations. Past medical history revealed hypertension well controlled with a single antihypertensive. There is no family history of any cardiac disease. Post-cardiac arrest ECG is normal (Figure 1) and his blood results were normal. Patient was shifted for computed tomography (CT) pulmonary angiography for pulmonary embolism (Figure 2) where on table he had a cardiac arrest that was cardioverted back to sinus rhythm.

**Figure 3:**
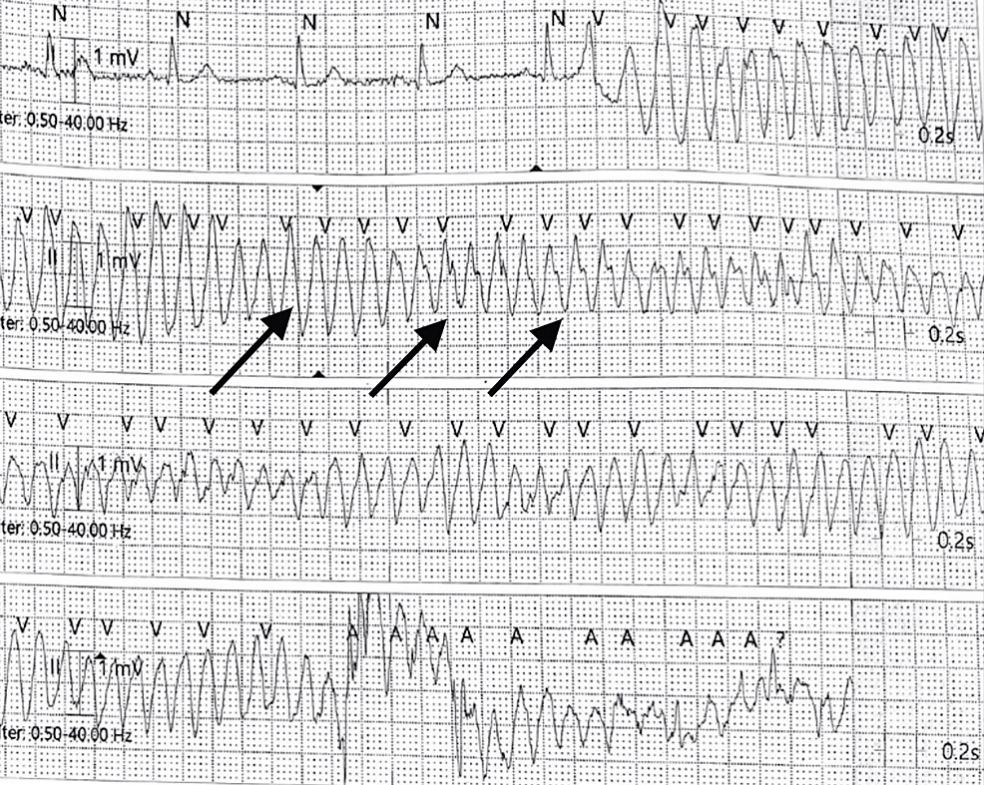
Rhythm strip during Coronary Care Unit stay showing monomorphic ventricular tachycardia (VT) (as depicted by arrows: absent P waves, broad QRS complexes of same morphology, regular RR interval)

Patient was commenced on bisoprolol, magnesium and amiodarone. The patient then was moved to the Coronary Care Unit because of the high risk and need for close monitoring. A rhythm strip (Figure 3) showed ventricular tachycardia in the Coronary Care Unit before he arrested again.

**Figure 4:**
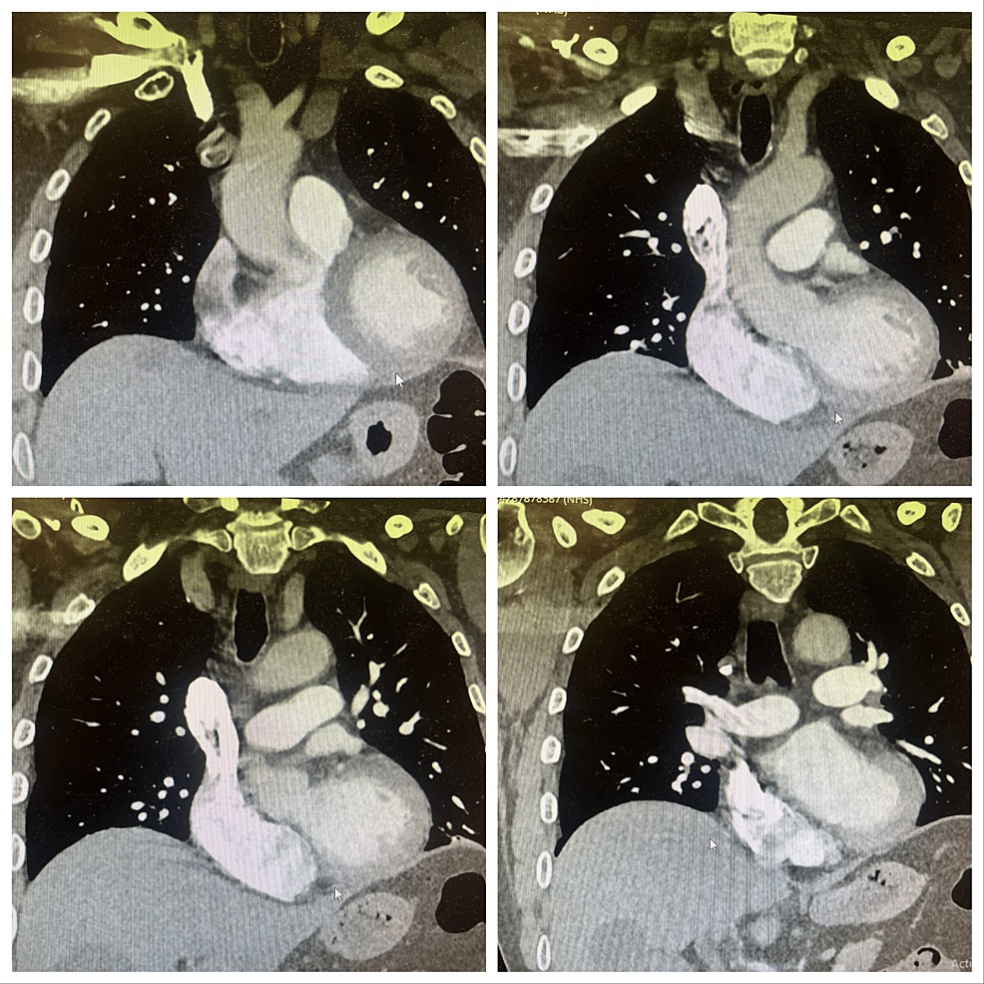
Cardiac magnetic resonance imaging (MRI) done to identify any infiltrative disease like amyloidosis/sarcoidosis

**Figure 5:**
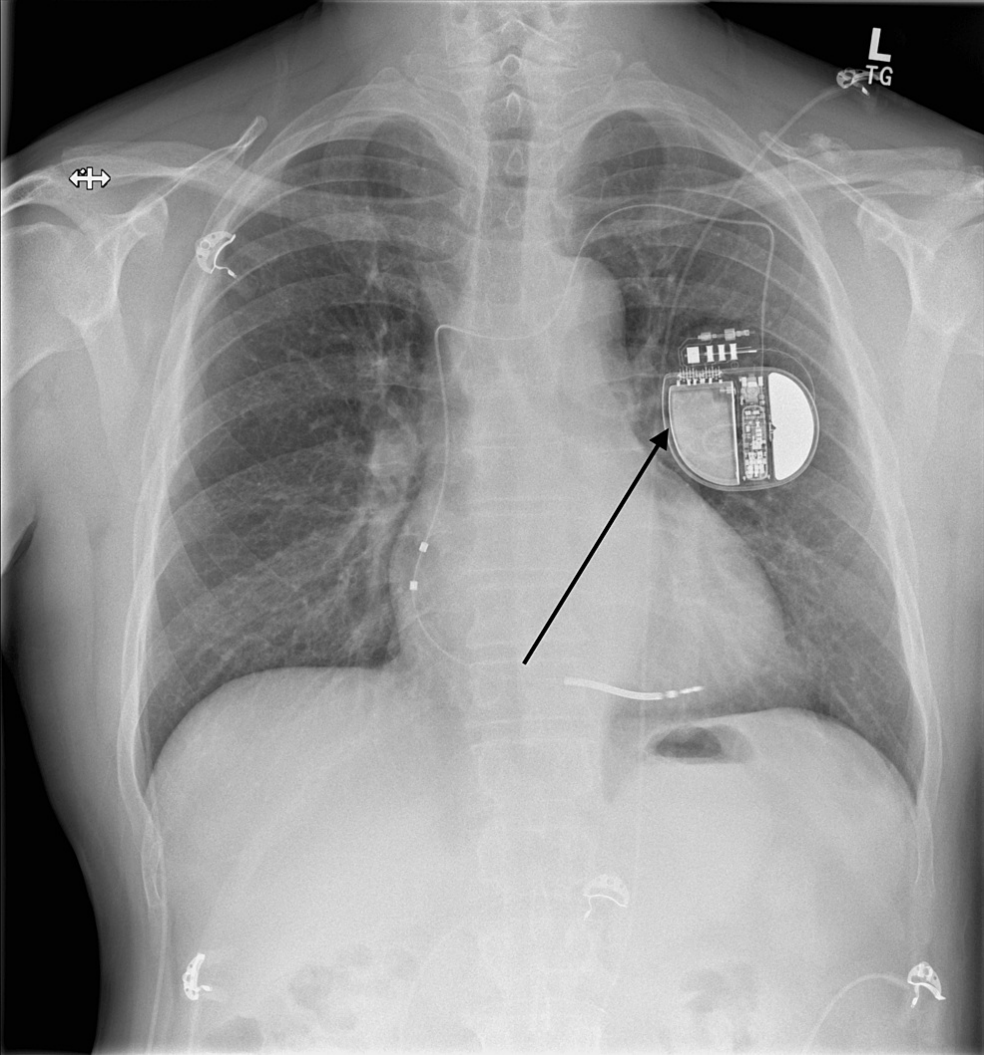
Chest X-ray post dual chamber implantable cardioverter defibrillator (ICD) implantation (arrows showing ICD on left side of chest)

Bedside echo was normal with no regional wall motion abnormalities and normal ejection fraction. He suffered another arrest with same outcome after which he was taken for angiography revealing patent vessels. He was commenced on two more antiarrhythmic drugs: esmolol and lignocaine infusion. His cardiac MRI (Figure 4) for structural heart disease was normal. He was hence discharged with implantable cardioverter defibrillator (ICD) implantation (Figure 5) after an arrest-free period for 24 hours. Genetic testing is currently being carried out to assess for an inherited cause of electrical storm.

## Discussion

Sudden cardiac arrest is a major public health problem with ventricular tachyarrythmias being the leading cause of sudden cardiac death. Out-of-hospital cardiac arrests in structurally normal hearts are rare with reported incidence less than 2% [7].

Electrical storm is defined as three or more distinct episodes of ventricular tachyarrythmias within a 24-hour period needing defibrillation input, or one or more episodes reoccurring within five minutes of termination [8]. The incidence of this storm varies between 10% and 28% in patients where ICD implantation was done as secondary prevention [9].

Patients with electrical storm present with a diverse range of symptoms like syncope, chest pain, palpitations or cardiac arrest [10]. Recent advances in comprehending the pathophysiology of this cardiac emergency and possible pharmacological therapy options have improved patient outcomes dramatically [11]. Pharmacological management with antiarrhythmic agents like amiodarone forms the cornerstone in managing patients with recurrent VT/ventricular fibrillation (VF) [12].

Like in our presented case, beta blockers need to be combined with antiarrhythmics to provide the best response and terminate the episode of VT/VF [13]. ICDs have now surpassed drug therapy as the first-line choice for prevention of sudden cardiac arrests in patients presenting with recurrent VT/VF [3].

## Conclusions

Electrical storms have been an enigma for a long time. Knowledge about resuscitation along with timely intervention with pharmacological therapy is of utmost importance. Our goal through this report is to share insights into managing electrical storm and the possible causes that can lead to this emergency.
